# Thai Massage, and Thai Herbal Compress versus Oral Ibuprofen in Symptomatic Treatment of Osteoarthritis of the Knee: A Randomized Controlled Trial

**DOI:** 10.1155/2014/490512

**Published:** 2014-09-01

**Authors:** Natthakarn Chiranthanut, Nutthiya Hanprasertpong, Supanimit Teekachunhatean

**Affiliations:** ^1^Department of Pharmacology, Faculty of Medicine, Chiang Mai University, Chiang Mai 50200, Thailand; ^2^Center of Thai Traditional and Complementary Medicine, Faculty of Medicine, Chiang Mai University, Chiang Mai 50200, Thailand

## Abstract

The aim of this study was to verify the clinical responses to Thai massage (TM) and Thai herbal compression (THC) for treating osteoarthritis (OA) of the knee in comparison to oral ibuprofen. This study was a randomized, evaluator-blind, controlled trial. Sixty patients with OA of the knee were randomly assigned to receive either a one-hour session of TM or THC (three times weekly) or oral ibuprofen (three times daily). The duration of treatment was three weeks. The clinical assessments included visual analog scale assessing pain and stiffness, Lequesne's functional index, time for climbing up ten steps, and physician's and patient's overall opinions on improvement. In a within-group comparison, each treatment modality caused a significant improvement of all variables determined for outcome assessments. In an among group comparison, all modalities provided nearly comparable clinical efficacy after a three-week symptomatic treatment of OA of the knee, in which a trend toward greatest improvement was likely to be found in THC group. In conclusion, TM and THC generally provided comparable clinical efficacy to oral ibuprofen after three weeks of treatment and could be considered as complementary and alternative treatments for OA of the knee.

## 1. Introduction

Osteoarthritis (OA) is considered to be the most common form of arthritis that results in structural and functional failure of synovial joints [1, 2]. The current paradigm holds that OA is a disease of the entire joint, involving not only the degradation of articular cartilage but also a variable subchondral bone reaction, and alteration of other joint structures, including the synovial membrane, meniscus, capsule, ligaments, and periarticular muscle [3]. The clinical features of OA include joint pain with activity, transient stiffness in the morning or after rest, restricted motion, joint crepitus, periarticular tenderness, bony swelling, and functional disability [3].

The knee is regarded as one of the most common joints frequently affected by OA [4]. The purposes of symptomatic treatment of OA of the knee are to control joint pain and to improve joint function [3]. The well-known pharmacological approach for symptomatic treatment includes oral administration of paracetamol, NSAIDs, opioids, and intra-articular corticosteroid injections [3, 4]. Although paracetamol should be prescribed as the preferred oral analgesic [5], it has been reported that the majority of patients with OA would prefer NSAIDs to paracetamol [6, 7]. However, NSAIDs should be used with caution in patients with peptic ulcer disease, renal insufficiency, or cardiovascular risk [8, 9]. Additionally, whereas opioids can be used for pain relief when NSAIDs have failed, their advantageous effects are outweighed by increased risk of adverse events and therefore should not be prescribed routinely [10]. Intra-articular corticosteroid injections should be considered in patients who do not respond satisfactorily to the aforementioned treatments as well as in patients with acute exacerbations of pain and signs of local inflammation [11]. In addition to these limitations of conventional pharmacological management, a number of patients with OA continue to experience inadequate pain control despite being treated accordingly [12]. These disadvantages lead many patients to seek for alternative health care options which are more or equally effective but less toxic than the conventional treatment.

Thai traditional massage is a Thai style procedure practiced on the human body using deep compressing, rhythmic pressing, and stretching. Thai traditional massage is purposed to relax muscle and tendon, increase body flexibility, improve range of joint motion, and improve regional blood circulation. Therefore, it offers a potentially beneficial role of relieving pain and improving functionality for many painful syndromes such as myofascial back pain [13], chronic low back pain [14], and muscle tension in patients with scapulocostal syndrome [15].

In addition to Thai traditional massage, Thai herbal compress is another traditional treatment and rehabilitation for painful syndromes. To conduct Thai herbal compress, various kinds of herbal ingredients with analgesic, anti-inflammatory, and muscle relaxant properties are mixed and tightly wrapped in a square piece of cloth to produce an herbal compress ball and then steamed in a stacked steamer pot. An application of warm compress ball to certain parts of the body enables active herbal ingredients (including aromatic oils) to permeate through the skin, leading to the therapeutic effects similar to those of Thai traditional massage mentioned above. Thai herbal compress is to be proven effective in musculoskeletal disorders including knee pain [16, 17].

Although both Thai traditional massage and Thai herbal compress are growing in popularity among Thai general public, the evidence-based data supporting their potential role for treating OA of the knee has been still limited and warrants further intensive investigation; this study aimed to explore the clinical responses and safety of both modalities in short-term symptomatic treatment of OA of the knee in comparison to oral ibuprofen, a nonsteroidal anti-inflammatory drug (NSAID).

## 2. Materials and Methods

### 2.1. Study Design

This study was a prospective, randomized, evaluator-blind, controlled study. The study was approved by the Human Research Ethics Committee of the Faculty of Medicine, Chiang Mai University, and complied with the Helsinki Declaration.

### 2.2. Subjects

The sample size calculation was based on the assumptions that mean change from baseline in VAS assessing total pain at the end of treatment was the main efficacy criterion, and the mean difference between a test treatment (*μ*_2_) and ibuprofen (*μ*_1_) was assumed to be 0 (i.e., *μ*_2_  (test) − *μ*_1_  (control) = 0). The noninferiority margin (*δ*) was chosen to be 60 points and the standard deviation (*σ*) was estimated to be 70. By using the following formula for noninferiority trial [18], the required sample size to achieve an 80% power (*β* = 0.2) at *α* = 0.05 for detecting such difference was 17 patients. With a projected dropout rate of 20%, twenty patients per treatment group were needed. Consider

(1) $$\begin{array}{c} n_{1}=n_{2}=\frac{2{\left(z_{\alpha }+z_{\beta }\right)}^{2}\sigma^{2}}{{\left(\mu_{2\;}-\;\mu_{1}-\delta \right)}^{2}}. \end{array}$$

Sixty out-patients of either sex were recruited. They were aged over 45 years and had been diagnosed with unilateral or bilateral OA of the knee according to the criteria of the American College of Rheumatology [19] for more than three months. After discontinuation of all OA treatment modalities over the run-in period of one week (week 0), the visual analog scale (VAS) assessing total pain had to be in the range of 175–375 out of 500. Participants had to be capable of walking. Signed informed consent was obtained prior to entry. Exclusion criteria included an underlying inflammatory arthropathy, gout, pseudogout, recent knee injury on the side affected by OA, expectation of knee arthroplasty in the near future, intra-articular corticosteroid injections within the previous three months, intolerance to NSAIDs, abnormal liver or kidney function tests, history of gastroduodenal ulcer and upper gastrointestinal hemorrhage, diabetes mellitus, poorly controlled hypertension, heart failure, pregnancy, nursing mother, and malignant tumors.

### 2.3. Treatment Procedures

**Figure 1:**
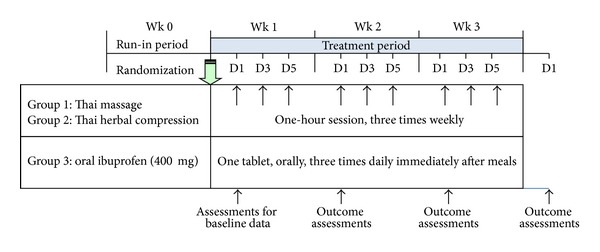
Schedule of treatment and assessment of this study. Clinical evaluation was performed on Day 1 (D1) of each week before receiving an assigned treatment or medication.

The study was conducted over a period of three weeks. Patients who met the eligible criteria were randomized by a computer-generated list into three treatment groups: Thai massage, Thai herbal compress, and ibuprofen group (Figure 1). The allocation sequence was carried out through placing the allocation cards in opaque, sealed, and stapled envelopes to preserve concealment. The envelopes were numbered in advance and opened sequentially when the patients met entry criteria and underwent randomization. During the entire study period, any other concurrent treatment modalities (including rescue analgesics) for the treatment of arthralgia and arthritis were not allowed.

#### 2.3.1. Thai Massage (TM)

**Figure 4:**
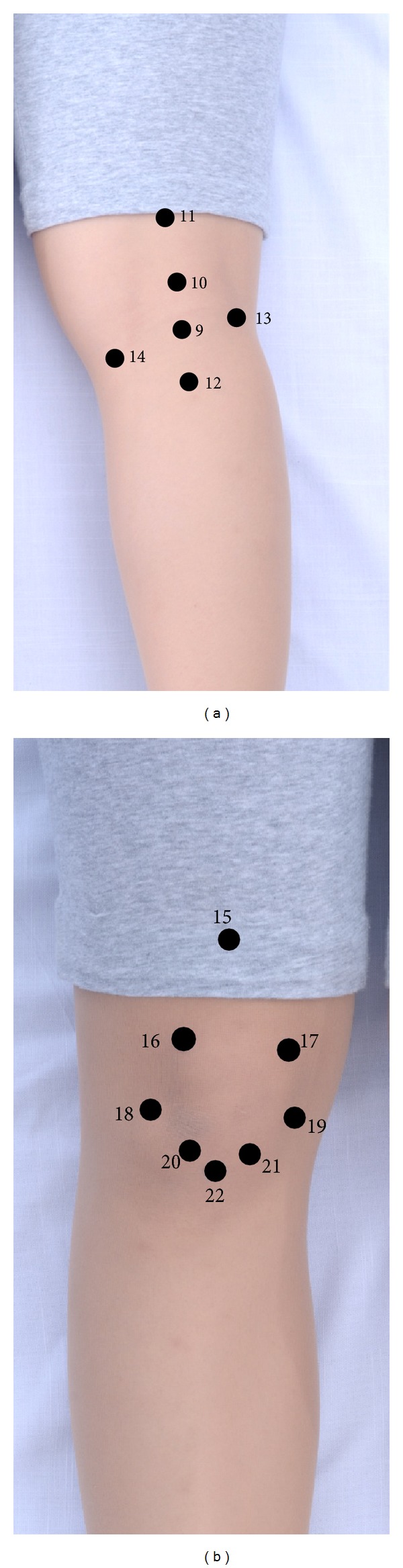
Pressure points located at the right knee; (a) posterior aspect and (b) anterior aspect.

The Thai massage technique selected in this study was “Suandok massage,” which is a low-risk and therapeutic-directed technique. Suandok massage has been recently developed by the research team at Faculty of Medicine, Chiang Mai University (also known locally as Suandok Hospital) in order to provide the Thai graceful massage procedures with minimized potential risks. In Suandok massage, some aggressive and potential harmful postures are thus excluded from fundamental procedures of Thai traditional massage such as compression using practitioner's knees or feet, trampling on the receiver's body, as well as fast and heavy twisting of the patient's body. Additionally, pressing onto specific Chinese acupressure points indicated for treatment of OA of the knee is also integrated in order to maximize effectiveness of Thai traditional massage. The aforementioned acupressure points included lateral and medial Xiyan (points 20 and 21, Figure 4), Heding (point 15, Figure 4), Weiyang (point 13, Figure 4), Weizhong (point 9, Figure 4), and Heyang (point 12, Figure 4). It is worth noting that these acupressure points frequently overlap with specific pressure points in Thai traditional massage.

Patients in TM group were assigned to receive Suandok massage on both lower extremities regardless of the affected side(s) of the knee, thirty minutes each side, three times a week on Monday, Wednesday, and Friday for three consecutive weeks. Main steps and methods of the massage are presented in Table 1. All patients in TM group received massage from the same professional practitioner who additionally underwent a 330-hour training course of Suandok massage held by Faculty of Medicine, Chiang Mai University. The commonly used technique in Suandok massage was the manipulation in which practitioner crossed two thumbs firmly and deeply pressed along body meridians including specific acupressure points. Compression to each point lasts about ten seconds.

#### 2.3.2. Thai Herbal Compression (THC)

Patients in THC group received application of herbal ball compress on both lower extremities. The duration, frequency, steps, and methods of THC were identical to those of TM, but the herbal ball was gently applied (simply touched without compression) along patient's meridians and upon acupressure points instead of manual manipulation as performed in Suandok massage. All patients in THC group received intervention from the same professional practitioner mentioned above.

Thai herbal compress balls were prepared by the Department of Pharmaceutical Sciences, Faculty of Pharmacy, Chiang Mai University. Each herbal compress ball weighed 225 g and contained dried herbs including* Zingiber cassumunar* Roxb. rhizomes (40%),* Curcuma longa* L. rhizomes (10%),* Cymbopogon citratus* (DC.) Stapf leaves and leaf sheaths (10%),* Croton roxburghii* N.P.Balakr. leaves (10%),* Tamarindus indica* L. leaves (10%),* Citrus hystrix* DC. peels (5%),* Blumea balsamifera* (L.) DC. leaves (5%),* Vitex trifolia* L. leaves (5%), and camphor (5%).

Before providing treatment on an individual patient, two herbal balls were steamed in a stacked steamer pot for twenty minutes. Afterwards, the first ball was wrapped with towel to protect the patient's skin from a burn due to excessive heat, and then the practitioner gently touched and rolled the herbal ball on the treated areas, approximately ten seconds for each point. Then, the towel was unwrapped when the ball was warm enough to put directly on the patient's body. The second ball was replaced when the first one was slightly lukewarm. The two herbal balls were alternately streamed and alternately used until each treatment session was achieved. After each session, the balls were wrapped in a plastic bag and kept in the freezer until reuse. This study allowed the reuse of herbal balls for three treatment visits. The new balls were replaced when the next round of treatment started.

#### 2.3.3. Ibuprofen

One 400 mg tablet of commercially marketed ibuprofen (Nurofen, Reckitt Benckiser Healthcare Manufacturing (Thailand) Limited) was prescribed, three times a day, immediately after meals for three weeks.

### 2.4. Assessments

Clinical assessments were evaluated at the end of a run-in period (week 0) for baseline data, and then weekly following each treatment for three consecutive weeks (week 1 to week 3) (Figure 1). These measured variables were as follows: (1) 100 mm VAS assessing pain over the last two days (classified into walking pain, standing pain, pain during climbing up and down stairs, night pain, resting pain, total pain, and pain during the most painful knee movement), of which 0 = no pain, 100 = severe pain; (2) 100 mm VAS assessing stiffness over the last two days (classified into morning stiffness, stiffness after rest, and total stiffness), of which 0 = no stiffness, be able to freely move, 100 = severe stiffness, very difficult to movement; (3) 100 mm VAS for physician's and patient's overall opinions of improvement over the last two days, of which 0 = no improvement, 100 = best possible improvement; (4) Lequesne's functional index assessing the patient's daily activities over the last two days (score ranging from 0–24) [20]; (5) time for climbing up ten steps. The participants self-rated the VAS and Lequesne's functional index, and they were allowed to view their own previously recorded scores. Additionally, at the end of the study period, the patients were considered as responders if their total pain score decreased at least by 80% in comparison to the baseline value [21]. Clinical assessments in each patient were evaluated by the same physician who was blinded to the treatment. Nondirective interviewing for adverse events and complete physical examination were also conducted weekly for three weeks in order to assess for safety.

### 2.5. Statistical Analysis

The statistical method in the present study was performed by an intention to treat analysis. Almost all datasets of outcome variables and their changes from baseline were proved to be normally distributed according to either Kolmogorov-Smirnov or Shapiro-Wilk test. In a within-group analysis, the mean values of VAS, Lequesne's functional index, and time for climbing up ten steps between baseline and the consecutive weeks were compared by the one-way analysis of variance (ANOVA) with repeated measurement.

In an among-group comparison, the one-way ANOVA was used to determine whether the three treatment groups differed in mean values of change from a baseline in VAS assessing pain and stiffness, Lequesne's functional index, as well as time for climbing up ten steps at the end of each week. Similarly, the mean values of VAS of the physician's and patient's overall opinions on improvement among the three groups at each particular time point were compared using the same test. When any statistical significance occurred between any of the three groups, the least significant difference (LSD) test was used to demonstrate statistical significance between each of the two groups. Differences among the treatment groups in number of patients considered as responders were evaluated by chi-square or Fisher's exact test.

## 3. Results

**Figure 5:**
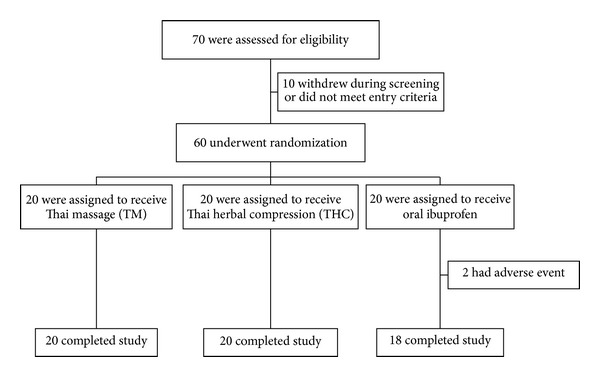
Flow chart of patients who participated in the clinical trial.

A total of 70 patients were enrolled into this study, of whom 10 were excluded (Figure 5). The remaining 60 patients were randomized into the TM, THC, and ibuprofen groups (20 patients per group). In the ibuprofen group, two patients withdrew from the study during the first week due to intolerance to the gastrointestinal adverse effects. The three treatment groups were not significantly different in baseline characteristics and baseline data for the major outcome assessment (VAS, Lequesne's functional index and time for climbing up ten steps) (Table 2). During the entire study period, the rates of adherence to treatment in the TM and THC group were 100%, whereas the rate of compliance with medication in the ibuprofen group was 90%.

In a within-group analysis (Tables 3 and 4), the mean values of every assessed parameter (i.e., VAS assessing pain and stiffness, Lequesne's functional index, and time for climbing up ten steps) in all groups were significantly improved compared with their own baselines. Notably, the improvement of all parameters reached statistical significance from the end of week 1, except for VAS assessing resting pain in TM group of which the significance was found since the end of week 2.

In an among-group analysis evaluated at the end of the study (Tables 5 and 6), the mean changes from baseline in most parameters did not differ among the three groups, except for the mean changes in VAS assessing pain during climbing up and down stairs in which statistical differences were found in favor of THC compared with ibuprofen and TM. Similarly, mean changes in Lequesne's functional index also significantly differed in favor of THC compared with ibuprofen. Nevertheless, it was worth noting that statistical differences among groups at the earlier time points were rarely found.

The mean values of VAS assessing physician's overall opinion of improvement were significantly different in favor of THC compared with TM at every time point (week 1–3), but there were no statistical significances between the remaining pairs of the three groups. On the other hand, the mean values of VAS assessing patient's overall opinion of improvement over the entire treatment period were generally comparable among the three groups, except for the end of week 1 in which THC group demonstrated significantly greater VAS than TM group (Table 7). On the basis of the number of responders whose VAS assessing total pain decreased at least by 80% compared with the baseline, the proportion of responders at the end of the study was comparable among the three groups (the response rate was 14 out of 20 patients or 70.0% in each group).

According to the reported adverse events, gastrointestinal adverse effects (nausea or abdominal pain) were significantly found in the ibuprofen group compared with TM and THC groups (8:0:0 events). Notably, two patients experienced severe abdominal pain and needed to be withdrawn from the study during the first week after initiation of oral ibuprofen. In addition, two events of skin rash and one event of edema were also found in ibuprofen group. In the TM group, three events of muscle aches over the treated area following the first massage session were reported. The adverse symptom was self-limited within a few days. In contrast, there was no reported adverse event in THC group.

## 4. Discussion

The results from this prospective, randomized, evaluator-blind, controlled study revealed that Thai massage, Thai herbal compress, and oral ibuprofen caused significant improvement of all parameters determined for outcome assessments. Additionally, all modalities provided nearly comparable clinical efficacy after a three-week symptomatic treatment of OA of the knee. The outcome parameters included in this study were consistent with recommendation of the Osteoarthritis Research Society International Standing Committee for Clinical Trials response criteria initiative, of which pain, function, and patient's global assessment were considered the main clinical variables to be included in OA clinical trials [22].

Usage of oral NSAID ibuprofen as controlled treatment in the present study was in agreement with the practice guidelines for OA recommended by the American College of Rheumatology [5]. An analgesic dose (1200 mg/day) of ibuprofen was reported to be as effective as an anti-inflammatory dose (2400 mg/day) [23] and also equivalent to anti-inflammatory doses of various NSAIDs such as indomethacin, phenylbutazone, and meclofenamate in relieving joint pain due to OA [24–26]. Gastrointestinal disorders are the well-known adverse events of NSAIDs, therefore it not surprising that more patients in the ibuprofen group experienced these unwanted effects and two patients were withdrawn due to intolerance to gastrointestinal adverse effects.

Cross-sectional studies have shown that arthritis is the most frequent reason for the elderly to use complementary and alternative medicine (CAM), probably due to ineffective pain relief, or adverse effects attributed to conventional medication, or the patients' own health beliefs [27]. So far, increasing interest has focused on massage therapy as complementary and alternative treatment for OA [28]. Nonetheless, since massage characteristics such as massage technique, duration, frequency, and number of sessions are supposed to closely relate to pain relief efficacy [29], the massage procedure used in this study was therefore performed by the same massage practitioner and confined to a one-hour session of Suandok massage, three times weekly for three consecutive weeks, in order to standardize massage therapy given to each patient in the TM group. A three times weekly dosing protocol was likely to be adequate according to the evidence from a previous randomized dose-finding trial showing that a one-hour once weekly massage protocol is the lowest optimal dose [30].

Several lines of scientific evidence support the effectiveness of massage in management of pain caused by musculoskeletal disorders [29, 31–35] including OA of the knee [36–38]. In the present study, a within-group analysis ascertained that TM appeared to be both statistically and clinically effective in symptomatic treatment of OA of the knee. These findings are in agreement with the previous studies demonstrating clinical efficacy of Thai massage (15-minute session, three times weekly for three weeks) [36], Swedish massage (one-hour session, twice weekly for four weeks, then once weekly for additional four weeks) [37], and self-massage (20-minute session, twice weekly during ten supervised and three unsupervised intervention sessions) [38] in patients with OA of the knee.

The potential underlying mechanisms of the action of massage remain unclear, but the proposed mechanisms probably include improving local blood flow; promoting venous circulation; increasing lymphatic drainage to remove waste products and reduce edema; improving the mobility of ligaments, tendons and muscle; as well as relaxing muscle tension [39]. Additionally, nerve transmission within large nerve fibers triggered by massage might also contribute to an analgesic effect via blocking the passage of painful stimuli entering through the spinal segment, which is known as the pain gate mechanism [40]. Another plausible analgesic mechanism is believed to mediate via descending inhibitory pain pathways, involving release of endogenous opioids within the spinal segment [41, 42]. Besides the aforementioned possibilities, an analgesic effect of massage in animal models is also demonstrated to exert via an endogenous release of oxytocin into the plasma and in the periaqueductal grey in the midbrain [43–45]. Oxytocin also causes an increase in beta-endorphin, L-encephalin, and dynorphin A1–13 contents in the rat spinal cord, suggesting the involvement of endogenous opiate peptide system in oxytocin-induced analgesia [46]. Notably, integration of Chinese acupressure into TM might produce additive or synergistic effects in management of OA of the knee since it has been found that five minutes of acupressure stimulation on the Xiyangguan acupoint, located on the lateral side of the knee joint, causes a significant increase in regional oxygen saturation of the deeper tissues on the same side of the knee in healthy volunteers. This study revealed that TM coupled with acupressure is an attractive alternative option in treatment of OA of the knee. Additionally, TM was quite safe; it involved a low incidence of mild muscle ache which was self-limited within a few days.

Topical heat therapy (such as hot pack, heat wrap therapy, localized microwave diathermy, and THC) is shown to be effective in increasing the range of joint motion, as well as in treatment of musculoskeletal pain [47–51]. The results reported in the present study were comparable with the previous findings demonstrating that THC, a simple and noninvasive traditional therapeutic procedure, could provide pain-reliving effects in OA of the knee [51].

In this study, THC was performed by gentle application without any compression of the herbal ball along meridians and upon acupressure points in order to ensure that clinical efficacy (if any) was not confounded by or attributed to pressing procedure that mimics TM. THC is proposed to exert its therapeutic effects via several possible mechanisms. First, these adventitious effects are most likely to be attributed to application of topical heat. Topical heat therapy triggers an increase in nerve conduction through small nonmyelinated C-fiber, which can inhibit pain signals entering through spinal segment [52]. Furthermore, an increase in temperature within skeletal muscles and soft tissue around the knees is postulated to be associated with an improvement of blood flow, leading to an elimination of inflammatory mediators from knee tissues [53]. Heat also improves connective tissue extensibility and range of joint motion, hence increases joint functionality [54]. Local application of heat is capable of improving the muscle fatigue characteristics [55]. In animal model, hot pack application can produce an augmented muscle force in exercised animals [56]. Additionally, hyperthermia results in an increased cellular level of heat shock proteins (HSPs), which are believed to mediate a protective effect against skeletal muscle damage [57]. Second, gentle application of herbal compress ball along the meridians and at acupressure points possibly also contributed to an analgesic effect. It was found that simple touch can produce significant improvements in immediate and sustained pain outcomes [58], probably via increased proprioception coupled with inhibited incoming pain signals (pain gate mechanism) [40]. Finally, permeation of various herbal constituents from herbal compress ball through tissues around the affected knee might play a crucial role for THC's therapeutic effects. Notably, analgesic and/or anti-inflammatory effects could be anticipated from the following constituents such as* Zingiber cassumunar* [59, 60],* Curcuma longa* [61, 62],* Cymbopogon citratus* [63–65],* Croton roxburghii* [66],* Tamarindus indica* [67],* Citrus hystrix* [68],* Blumea balsamifera* [69, 70],* Vitex trifolia* [71], and camphor [72].

In an among-group comparison, all three modalities provided nearly comparable clinical efficacy after a three-week symptomatic treatment of OA of the knee. Nevertheless, a trend toward greatest improvement, determined by magnitude of changes in most outcome variables, was likely to be found in THC group. Furthermore there were statistically better physician's overall opinion of improvement in favor of THC compared to TM, and significantly better improvement of Lequesne's functional index in favor of THC compared to ibuprofen, whereas no adverse event was reported in THC group. These findings suggest that THC should be an attractive alternative option in symptomatic treatment of OA of the knee, in comparison to TM or oral ibuprofen. It is unclear whether combination of TM and THC, as commonly seen in real situation of traditional practices, would contribute to better clinical outcomes. This interesting issue warrants further investigation.

Some limitations regarding this study should be mentioned. The time course of clinical improvement caused by TM and THC seems to gradually accumulate over the study period and it could not ensure whether or not maximal efficacy had already been achieved at the end of week 3. Further study with a longer study period should be pursued. Additionally, there was no posttreatment follow-up to evaluate the carryover effect produced by each treatment modality, especially TM and THC. Actually, the efficacy of massage sessions on pain relief has been shown to last several more weeks despite discontinuation of treatment [73]. Similarly, beneficial effects of deep heating therapy via microwave diathermy (three 30-minute sessions a week for four weeks) on improvement of pain, muscle strength, and physical function in patients with moderate OA of the knee have been demonstrated to be sustained over 12 months of follow-up [49]. This potential carryover benefit should be further investigated. Another limitation that each assigned treatment was unable to be adequately blinded to the patients, possibly resulted in bias. Lastly, a small sample size of this study might contribute to an inadequate power to differentiate the significant difference in clinical efficacy among groups (if any). Therefore, further study using a sufficiently large sample size is warranted.

## 5. Conclusion

TM, THC, and oral ibuprofen caused a significant improvement of all variables determined for outcome assessments. All modalities provided nearly comparable clinical efficacy after a three-week symptomatic treatment of OA of the knee, in which a trend toward greatest improvement was likely to be found in THC group. TM and THC were generally safe and free from systemic adverse effects. Both modalities could be therefore considered as effective alternative options for treatment of OA of the knee, especially in individuals who do not wish to receive oral NSAIDs or who experienced systemic unwanted effects from oral NSAIDs.

## Figures and Tables

**Figure 2:**
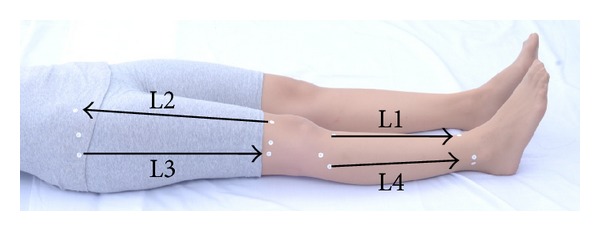
Meridians (L1–L4 energy lines) on the right lower extremity in the supine position.

**Figure 3:**
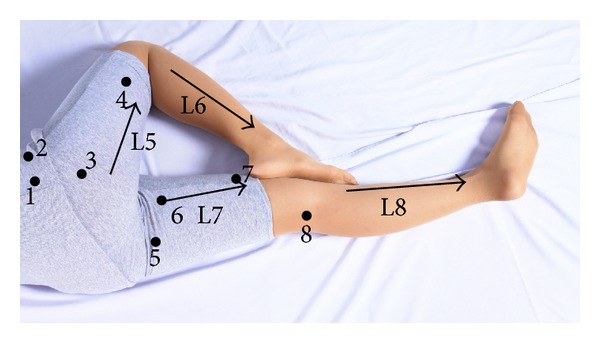
Meridians and pressure points on lateral aspect of the right lower extremity and medial aspect of the left lower extremity in the left lateral recumbent position.

**Table 1:** Main steps and methods of Suandok massage used in this study.

	Step of massage	Method
1	Massage on the right lower extremity in the supine position (Figure 2)	Massage practitioner kneeled at the right side of the patient, started thumb pressing at the right lower extremity along L1–L4 meridians, respectively, and then crossed the two hands with palm down to press on groin to “open the wind gate” for thirty seconds.

2	Massage on lateral aspect of the right lower extremity and medial aspect of the left lower extremity in the left lateral recumbent position (Figure 3)	Massage practitioner kneeled behind the patient, started thumb pressing at the three points (point numbers 1–3) located at right gluteal region, and continued thumb pressing at posterior aspect of the right thigh along L5 meridian and at the point number 4, respectively. Next, crossed the two hands with palm down to press at posterolateral aspect of the right lower leg along L6 meridian and finished with massage on the right foot. Afterwards, switched to massage on the left lower extremity by crossing the two hands with palm down to press at medial aspect of the left thigh along L7 meridian, then performed thumb pressing at the four points located at the left thigh and at popliteal fossa (point numbers 5–8), and continued thumb pressing at medial aspect of the left lower leg along L8 meridian, and then finished with massage on left foot.

3	Massage on the left lower extremity in the supine position	Followed step 1, but performed on the left lower extremity.

4	Massage on lateral aspect of the left lower extremity and medial aspect of the right lower extremity in the right lateral recumbent position	Followed step 2, but performed on the left lower extremity.

5	Massage on posterior aspect of both lower extremities in the prone position and massage on the posterior aspect of both knees (Figure 4(a))	Massage practitioner kneeled at the right side of the patient; started massage from both feet up to upper hips along posterior aspect of both extremities and then pressed on six points located at the posterior aspect of both knees (point numbers 9–14).

6	Massage on the anterior aspect of both knees in the supine position (Figure 4(b))	Massage practitioner kneeled at the right side of the patient and pressed on eight points located above and around the patella of both knees (point numbers 15–22).

**Table 2:** Demographic characteristics and baseline data for the major outcome assessments of participants evaluated at the end of a run-in period (week 0).

Characteristics	Treatment groups			*P* value
	TM	THC	Ibuprofen	
*n* (male: female)	20 (6 : 14)	20 (5 : 15)	20 (5 : 15)	0.918
Age (y)^§^	65.45 ± 9.75	63.70 ± 6.07	62.25 ± 9.50	0.504
Body weight (kg)^§^	65.37 ± 19.92	65.00 ± 12.86	62.37 ± 10.32	0.787
Height (m)^§^	1.53 ± 0.08	1.55 ± 0.08	1.54 ± 0.07	0.749
Body mass index (kg/m^2^)^§^	27.78 ± 8.20	26.76 ± 3.56	26.30 ± 4.56	0.712
Duration of OA (y)^§^	7.37 ± 7.16	6.45 ± 4.87	7.95 ± 8.20	0.787
Localization of OA				0.893
Right knee	1	2	2	
Left knee	4	2	3	
Both knees	15	16	15	
Kellgren and Lawrence X-ray grade (knee)				
Grade 2	12	11	17	0.461
Grade 3	13	14	11	0.832
Grade 4	10	11	7	0.629
VAS assessing pain (mm)^§^				
Walking pain	58.05 ± 13.48	61.40 ± 17.94	53.35 ± 13.70	0.250
Standing pain	55.10 ± 13.29	54.85 ± 19.80	52.50 ± 18.84	0.875
Pain during climbing up and down stairs	55.80 ± 20.75	62.80 ± 21.22	58.20 ± 22.37	0.580
Night pain	54.60 ± 20.35	40.00 ± 26.28	50.20 ± 19.81	0.115
Resting pain	35.65 ± 18.83	41.20 ± 21.88	39.50 ± 18.81	0.667
Total pain^a^	259.20 ± 56.20	260.25 ± 68.86	253.75 ± 63.45	0.941
Pain during the most painful knee movement	74.60 ± 14.34	76.70 ± 14.94	76.95 ± 14.07	0.853
VAS assessing stiffness (mm)^§^				
Morning stiffness	59.15 ± 22.88	51.30 ± 30.89	54.00 ± 23.79	0.629
Stiffness after rest	53.80 ± 22.16	49.80 ± 26.86	53.10 ± 24.17	0.859
Total stiffness^b^	112.95 ± 41.04	101.10 ± 55.42	107.10 ± 44.75	0.733
Lequesne's functional index^§^	13.50 ± 2.38	13.25 ± 4.07	12.70 ± 2.41	0.700
Time for climbing up ten steps^§^	11.80 ± 4.26	13.55 ± 5.61	13.15 ± 6.57	0.584

^§^Data represent mean ± SD.

^a^Summation of VAS that assessed walking pain, standing pain, pain during climbing up and down stairs, night pain, and resting pain.

^b^Summation of VAS that assessed morning stiffness and stiffness after rest.

**Table 3:** Mean VAS assessing pain (mm) in intent-to-treat patients (*n* = 20/group).

Variable	Treatment group	Week 0	Week 1	Week 2	Week 3
VAS assessing pain (mm)					
Walking pain	TM	58.05 ± 13.48	42.45 ± 15.02∗∗∗	26.60 ± 12.19∗∗∗	17.70 ± 15.01∗∗∗
	THC	61.40 ± 17.94	39.00 ± 18.91∗∗∗	26.70 ± 16.66∗∗∗	16.35 ± 17.54∗∗∗
	Ibuprofen	53.35 ± 13.70	36.40 ± 16.48∗∗	28.75 ± 16.47∗∗∗	19.85 ± 17.96∗∗∗
Standing pain	TM	55.10 ± 13.29	38.25 ± 20.88∗∗∗	26.90 ± 19.15∗∗∗	20.05 ± 21.69∗∗∗
	THC	54.85 ± 19.80	40.75 ± 19.19∗∗	26.35 ± 17.81∗∗∗	16.70 ± 15.69∗∗∗
	Ibuprofen	52.50 ± 18.84	35.45 ± 16.89∗∗	26.85 ± 18.63∗∗∗	19.80 ± 17.84∗∗∗
Pain during climbing up and down stairs	TM	55.80 ± 20.75	45.50 ± 22.19∗∗	31.75 ± 20.26∗∗∗	23.65 ± 16.91∗∗∗
	THC	62.80 ± 21.22	42.50 ± 18.03∗∗∗	29.00 ± 15.40∗∗∗	14.20 ± 11.52∗∗∗
	Ibuprofen	58.20 ± 22.37	43.95 ± 22.36∗∗	32.90 ± 22.99∗∗∗	25.25 ± 21.76∗∗∗
Night pain	TM	54.60 ± 20.35	32.75 ± 19.25∗∗∗	19.10 ± 21.40∗∗∗	14.60 ± 20.57∗∗∗
	THC	40.00 ± 26.28	27.40 ± 23.25∗∗	16.00 ± 19.16∗∗∗	5.75 ± 9.16∗∗∗
	Ibuprofen	50.20 ± 19.81	29.80 ± 16.58∗∗∗	18.55 ± 20.05∗∗∗	14.20 ± 18.13∗∗∗
Resting pain	TM	35.65 ± 18.83	26.80 ± 16.59	15.20 ± 15.26∗∗	11.95 ± 14.46∗∗
	THC	41.20 ± 21.88	22.45 ± 19.87∗∗	11.10 ± 14.28∗∗∗	8.55 ± 11.07∗∗∗
	Ibuprofen	39.50 ± 18.81	24.60 ± 17.39∗∗∗	20.05 ± 16.12∗∗	9.80 ± 13.92∗∗∗
Total pain^a^	TM	259.20 ± 56.20	185.75 ± 67.31∗∗∗	119.55 ± 71.98∗∗∗	87.95 ± 75.81∗∗∗
	THC	260.25 ± 68.86	172.10 ± 73.42∗∗∗	109.15 ± 61.56∗∗∗	61.55 ± 49.42∗∗∗
	Ibuprofen	253.75 ± 63.45	170.20 ± 62.85∗∗∗	107.40 ± 82.15∗∗∗	69.20 ± 71.02∗∗∗
Pain during the most painful knee movement	TM	74.60 ± 14.34	59.05 ± 17.89∗∗	45.50 ± 24.66∗∗∗	31.15 ± 22.30∗∗∗
	THC	76.70 ± 14.94	48.35 ± 25.70∗∗∗	28.60 ± 20.24∗∗∗	22.60 ± 19.91∗∗∗
	Ibuprofen	76.95 ± 14.07	49.90 ± 23.72∗∗∗	36.20 ± 24.71∗∗∗	29.55 ± 25.60∗∗∗

Data represent mean ± SD. ^a^Summation of VAS that assessed walking pain, standing pain, pain during climbing up and down stairs, night pain, and resting pain. ∗*P *< 0.05, ∗∗*P* < 0.01, ∗∗∗*P* < 0.001 versus baseline.

**Table 4:** Mean values of VAS assessing stiffness, Lequesne's functional index, and time for climbing up ten steps in intent-to-treat patients (*n* = 20/group).

Variable	Treatment group	Week 0	Week 1	Week 2	Week 3
VAS assessing stiffness (mm)					
Morning stiffness	TM	59.15 ± 22.88	41.90 ± 22.63∗∗∗	29.00 ± 20.89∗∗∗	16.65 ± 16.68∗∗∗
	THC	51.30 ± 30.89	34.40 ± 21.26∗∗	16.80 ± 14.24∗∗∗	5.05 ± 6.30∗∗∗
	Ibuprofen	54.00 ± 23.79	33.10 ± 21.67∗∗	23.25 ± 20.44∗∗∗	15.60 ± 19.56∗∗∗
Stiffness after rest	TM	53.80 ± 22.16	36.85 ± 20.58∗∗∗	23.60 ± 17.97∗∗∗	19.95 ± 19.04∗∗∗
	THC	49.80 ± 26.86	30.70 ± 22.29∗∗∗	17.85 ± 14.50∗∗∗	10.65 ± 12.49∗∗∗
	Ibuprofen	53.10 ± 24.17	34.70 ± 19.16∗∗	24.10 ± 20.76∗∗∗	16.30 ± 18.28∗∗∗
Total stiffness^a^	TM	112.95 ± 41.04	78.75 ± 38.88∗∗∗	52.60 ± 36.50∗∗∗	36.60 ± 32.22∗∗∗
	THC	101.10 ± 55.42	65.10 ± 41.00∗∗∗	34.65 ± 28.35∗∗∗	15.70 ± 15.85∗∗∗
	Ibuprofen	107.10 ± 44.75	67.80 ± 37.47∗∗	47.35 ± 38.82∗∗∗	31.90 ± 36.28∗∗∗
Lequesne's functional index (score)	TM	13.50 ± 2.38	11.18 ± 3.13∗∗∗	9.40 ± 3.23∗∗∗	7.73 ± 3.30∗∗∗
	THC	13.25 ± 4.07	10.68 ± 3.54∗∗	8.30 ± 2.93∗∗∗	6.53 ± 3.73∗∗∗
	Ibuprofen	12.70 ± 2.41	10.20 ± 2.59∗∗∗	8.35 ± 3.05∗∗∗	7.85 ± 3.31∗∗∗
Time for climbing up ten steps (second)	TM	11.80 ± 4.26	10.50 ± 3.87∗	9.10 ± 3.24∗∗	8.25 ± 3.08∗∗∗
	THC	13.55 ± 5.61	10.70 ± 6.34∗	8.90 ± 3.24∗∗∗	7.95 ± 2.78∗∗∗
	Ibuprofen	13.15 ± 6.57	11.55 ± 6.23∗∗	10.00 ± 4.41∗∗	9.25 ± 3.61∗∗∗

Data represent mean ± SD. ^a^Summation of VAS that assessed morning stiffness and stiffness after rest. ∗*P *< 0.05, ∗∗*P* < 0.01, ∗∗∗*P* < 0.001 versus baseline.

**Table 5:** Mean changes from baseline in VAS assessing pain in intent-to-treat patients (*n* = 20/group).

Variable	Treatment group	Week 0-1	Week 0–2	Week 0–3
VAS assessing pain (mm)				
Walking pain	TM	−15.60 ± 15.25	−31.45 ± 13.33	−40.35 ± 15.55
	THC	−22.40 ± 14.09	−34.70 ± 18.70	−45.05 ± 18.76
	Ibuprofen	−16.95 ± 18.35	−24.60 ± 22.83	−33.50 ± 23.80
Standing pain	TM	−16.85 ± 17.28	−28.20 ± 14.06	−35.05 ± 16.32
	THC	−14.10 ± 15.21	−28.50 ± 21.54	−38.15 ± 20.25
	Ibuprofen	−17.05 ± 20.01	−25.65 ± 23.39	−32.70 ± 26.35
Pain during climbing up and down stairs	TM	−10.30 ± 11.07	−24.05 ± 17.40	−32.15 ± 19.01
	THC	−20.30 ± 15.84^†^	−33.80 ± 21.20	−48.60 ± 19.45^∗,†^
	Ibuprofen	−14.25 ± 17.14	−25.30 ± 23.59	−32.95 ± 26.07
Night pain	TM	−21.85 ± 14.80	−35.50 ± 24.59	−40.00 ± 25.16
	THC	−12.60 ± 16.48	−24.00 ± 21.68	−34.25 ± 25.32
	Ibuprofen	−20.40 ± 19.73	−31.65 ± 23.72	−36.00 ± 23.40
Resting pain	TM	−8.85 ± 21.59	−20.45 ± 24.80	−23.70 ± 27.53
	THC	−18.75 ± 26.76	−30.10 ± 24.60	−32.65 ± 24.10
	Ibuprofen	−14.90 ± 12.86	−19.45 ± 21.22	−29.70 ± 24.02
Total pain^a^	TM	−73.45 ± 38.70	−139.65 ± 63.68	−171.25 ± 71.94
	THC	−88.15 ± 54.65	−151.10 ± 70.92	−198.70 ± 71.19
	Ibuprofen	−83.55 ± 64.06	−146.35 ± 94.02	−184.55 ± 92.36
Pain during the most painful knee movement	TM	−15.55 ± 19.69	−29.10 ± 25.14	−43.45 ± 23.00
	THC	−28.35 ± 19.00	−48.10 ± 18.52^†^	−54.10 ± 17.94
	Ibuprofen	−27.05 ± 21.85	−40.75 ± 23.97	−47.40 ± 26.85

Data represent mean ± SD. ^a^Summation of VAS that assessed walking pain, standing pain, pain during climbing up and down stairs, night pain, and resting pain. ∗Statistical significance versus ibuprofen (*P *= 0.027). ^†^Statistical significance versus TM (*P *= 0.038 and *P *= 0.020; pain during climbing up and down stairs at week 0-1 and 0–3, respectively, *P *= 0.011; pain during the most painful knee movement at week 0–2).

**Table 6:** Mean changes from baseline in VAS assessing stiffness, Lequesne's functional index, and time for climbing up ten steps in intent-to-treat patients (*n* = 20/group).

Variable	Treatment group	Week 0-1	Week 0–2	Week 0–3
VAS assessing stiffness (mm)				
Morning stiffness	TM	−17.25 ± 17.99	−30.15 ± 21.86	−42.50 ± 21.97
	THC	−16.90 ± 21.84	−34.50 ± 28.86	−46.25 ± 29.68
	Ibuprofen	−20.90 ± 23.68	−30.75 ± 29.94	−38.40 ± 31.44
Stiffness after rest	TM	−16.95 ± 16.37	−30.20 ± 19.40	−33.85 ± 25.12
	THC	−19.10 ± 19.81	−31.95 ± 23.53	−39.15 ± 23.78
	Ibuprofen	−18.40 ± 23.25	−29.00 ± 30.25	−36.80 ± 29.47
Total stiffness^a^	TM	−34.20 ± 29.54	−60.35 ± 38.37	−76.35 ± 41.56
	THC	−36.00 ± 36.45	−66.45 ± 48.24	−85.40 ± 49.46
	Ibuprofen	−39.30 ± 44.51	−59.75 ± 59.42	−75.20 ± 59.45
Lequesne's functional index (score)	TM	−2.33 ± 2.30	−4.10 ± 3.01	−5.78 ± 2.41
	THC	−2.58 ± 2.82	−4.95 ± 3.45	−6.73 ± 3.45∗
	Ibuprofen	−2.50 ± 2.33	−4.35 ± 2.47	−4.85 ± 2.84
Time for climbing up ten steps (second)	TM	−1.30 ± 2.52	−2.70 ± 3.15	−3.55 ± 3.33
	THC	−2.85 ± 4.64	−4.65 ± 4.08	−5.60 ± 4.57
	Ibuprofen	−1.60 ± 1.93	−3.15 ± 3.42	−3.90 ± 4.00

Data represent mean ± SD. ^a^Summation of VAS that assessed morning stiffness and stiffness after rest.

∗Statistical significance versus ibuprofen (*P *= 0.048).

**Table 7:** 100-mm VAS assessing physician's and patient's overall opinions of improvement evaluated during treatment.

Variable	Treatment group	*n*	Week 1	Week 2	Week 3
Patient's overall opinion (mm)^§^	TM	20	38.55 ± 20.46	60.00 ± 19.72	77.60 ± 16.01
	THC	20	57.10 ± 17.70^†^	72.15 ± 17.82	85.65 ± 11.77
	Ibuprofen	18^*π*^	51.70 ± 26.59	65.11 ± 25.07	78.11 ± 23.73

Physician's overall opinion (mm)^§^	TM	20	26.35 ± 9.89	36.90 ± 10.80	51.75 ± 10.83
	THC	20	42.75 ± 17.64^††^	56.70 ± 15.89^††^	66.05 ± 13.15^††^
	Ibuprofen	18^*π*^	33.90 ± 20.05	46.50 ± 24.68	55.28 ± 24.20

Data represent mean ± SD.

^§^0 = no improvement, 100 = best possible improvement.

^*π*^2 patients in the ibuprofen group could not be assessed due to being withdrawn during week 1.

^†^Statistical significance versus TM (*P *= 0.010).

^††^Statistical significance versus TM (*P *= 0.003 at week 1, *P *= 0.001 at week 2, and *P *= 0.009 at week 3).
